# Inoculation of tomato plants with rhizobacteria enhances the performance of the phloem-feeding insect *Bemisia tabaci*

**DOI:** 10.3389/fpls.2013.00306

**Published:** 2013-08-13

**Authors:** Roee Shavit, Maya Ofek-Lalzar, Saul Burdman, Shai Morin

**Affiliations:** ^1^Department of Entomology, The Robert H. Smith Faculty of Agriculture, Food and Environment, The Hebrew University of JerusalemRehovot, Israel; ^2^Department of Plant Pathology and Microbiology, The Robert H. Smith Faculty of Agriculture, Food and Environment, The Hebrew University of JerusalemRehovot, Israel; ^3^Department of Soil, Water and Environmental Sciences, Agricultural Research Organization of IsraelBet Dagan, Israel

**Keywords:** *Bemisia tabaci*, induced systemic resistance, plant growth promoting rizhobacteria, plant signaling, generalist phloem-feeders

## Abstract

In their natural environment, plants experience multiple biotic interactions and respond to this complexity in an integrated manner. Therefore, plant responses to herbivory are flexible and depend on the context and complexity in which they occur. For example, plant growth promoting rhizobacteria (PGPR) can enhance plant growth and induce resistance against microbial pathogens and herbivorous insects by a phenomenon termed induced systemic resistance (ISR). In the present study, we investigated the effect of tomato (*Solanum lycopersicum*) pre-inoculation with the PGPR *Pseudomonas fluorescens* WCS417r, on the performance of the generalist phloem-feeding insect *Bemisia tabaci*. Based on the ability of *P. fluorescens* WCS417r to prime for ISR against generalists chewing insects and necrotrophic pathogens, we hypothesized that pre-inoculated plants will strongly resist *B. tabaci* infestation. In contrast, we discovered that the pre-inoculation treatment increased the tomato plant suitability for *B. tabaci* which was emphasized both by faster developmental rate and higher survivability of nymph stages on pre-inoculated plants. Our molecular and chemical analyses suggested that the phenomenon is likely to be related to: (I) the ability of the bacteria to reduce the activity of the plant induced defense systems; (II) a possible manipulation by *P. fluorescens* of the plant quality (in terms of suitability for *B. tabaci*) through an indirect effect on the rhizosphere bacterial community. The contribution of our study to the pattern proposed for other belowground rhizobacteria and mycorrhizal fungi and aboveground generalist phloem-feeders is discussed.

## Introduction

Plants rely both on constitutive and induced defenses in order to achieve optimal protection against their herbivorous enemies (Baldwin, 2001; Howe and Jander, 2008; Wu and Baldwin, 2010). These defenses can influence herbivore settling, feeding, oviposition, growth and development, fecundity, and/or fertility (Baldwin and Preston, 1999; Walling, 2000; Elsayed, 2011). Plant induced defenses involve activation of distinct signal-transduction pathways, in which three major plant hormones—salicylic acid (SA), jasmonic acid (JA) and ethylene (ET) are involved (Glazebrook, 2005; Bari and Jones, 2009; Pieterse et al., 2009).

The SA pathway is primarily activated in response to biotrophic pathogens, and regulates the expression of a wide array of defense-responses including the *Pathogenensis-related protein* (PR) coding genes (Ward et al., 1991; Van Loon and Van Strien, 1999; Van Loon et al., 2006). The JA/ET pathways are induced in response to necrotrophic pathogens and in response to wounding and tissue-damaging by insect feeding (Kessler and Baldwin, 2002; Kaloshian and Walling, 2005; Bari and Jones, 2009). JA and ET can either cooperate or act as antagonists in the regulation of different stress responses (i.e., to pathogen attack or wounding) (Lorenzo and Solano, 2005). In the case of necrotrophic pathogens, both hormones cooperate or synergize in the activation of defense gene expression (Lorenzo et al., 2003). However, in the case of wound response, an antagonistic interaction between JA and ET has been described (Rojo et al., 1999). Two transcription factors, ERF1 and MYC2, have been shown to participate in the regulation of these interactions. ERF1 is induced by simultaneous action of the JA and ET signaling pathways, and plays a key role in the activation of plant defenses against necrotrophic pathogen infection by regulating defense-related genes, such as *PDF1.2* (Lorenzo et al., 2003; Kazan and Manners, 2008). MYC2, on the other hand, is positively regulated by the abscisic acid (ABA) signaling pathway and functions as a transcriptional activator of genes (for example *VSP2* in *A. thaliana*) in the MYC2-branch of the JA pathway, which is associated with plant responses to wounding or insect herbivory (Lorenzo et al., 2004; Dombrecht et al., 2007). Examples of JA/ET inducible proteins that have an established or putative role in direct plant defenses include: proteinase inhibitor (PI), polyphenol oxidase (PPO), arginase, threonine deaminase, leucine amino peptidase and acid phosphatase (Walling, 2000; Howe and Jander, 2008). SA and JA/ET signaling pathways can also mutually affect each other largely through negative cross-talk. This cross-talk helps the plant to minimize fitness costs and creates a flexible signaling network that allows the plant to fine-tune its defense response to the corresponding invaders (Kunkel and Brooks, 2002; Bostock, 2005).

Two forms of induced resistance that are systemically expressed in the plant, and effective against microbial pathogens and insect herbivores have been well-characterized: systemic acquired resistance (SAR), which is typically activated upon primary limited infection by a plant pathogen (Durrant and Dong, 2004); and induced systemic resistance (ISR), which is typically elicited by specific strains of non-pathogenic plant growth-promoting rhizobacteria (PGPR) or fungi (Van Loon et al., 1998). SAR is controlled by a signaling pathway that depends on endogenous accumulation of SA and the defense regulatory protein NPR1 (Durrant and Dong, 2004). As stated above, SAR is associated with the activation of *PR* genes, some of which encode proteins with antimicrobial activity (Van Loon et al., 2006). On the other hand, the onset of ISR is not associated with enhanced expression of defense-related genes (Verhagen et al., 2004). In contrast, ISR is characterized by the establishment of a primed state for defense, in which defense-related responses are induced more rapidly upon pathogen or insect attack, thereby leading to a metabolically less costly state of resistance (Heil and Walters, 2009). The molecular mechanisms of ISR have been especially studied in *Arabidopsis thaliana* triggered by the rhizobacterium *Pseudomonas fluorescens* WCS417r, which indicated that PGPR-mediated ISR is often associated with increased callose deposition at the site of pathogen entry and enhanced expression of JA/ET-responsive genes, such as *PDF1.2* and *VSP2* (Pieterse et al., 1996; Ahn et al., 2007; Van Oosten et al., 2008).

As JA is a key regulator of both ISR and plant defenses against herbivorous insects, it has been speculated that ISR induced by PGPR should benefit plants in their battle against insect herbivores. Indeed, studies with *A. thaliana* plants that were pre-inoculated with the PGPR *P. fluorescens* WCS417r showed that the growth and development of the generalist caterpillar *Spodoptera exigua* was negatively affected by the PGPR treatment. However, the negative effect of the PGPR-induced ISR was found not to be a general one, as no differences were observed between pre-inoculated and non-inoculated treatments in the growth and development of the specialist caterpillar *Pieris rapae* subjected to the same plant-PGPR system (Van Oosten et al., 2008). Recently, an additional level of complexity was added to our understanding of the rhizobacteria—herbivore insect systems as it was found that *P. fluorescens* WCS417r has a positive effect on the performance (weight gain and intrinsic rate of increase) of the generalist phloem-feeding aphid *Myzus persicae* on *A. thaliana* (Pineda et al., 2012). The same authors indicated that the observed aforementioned differential effects of rhizobacteria on herbivores, are in line with trends reported for mycorrhizal fungi (Gehring and Bennett, 2009; Koricheva et al., 2009), which were similarly shown to be dependent on the feeding-guild: negative effects on the development of generalist leaf-chewing insects and mesophyll feeders, neutral effects on specialist chewing and phloem-feeding insects, and un-predicted effects (negative, positive or neutral) on generalists phloem-feeding herbivores (De Vos et al., 2007a; Pineda et al., 2010; Valenzuela-Soto et al., 2010; Pineda et al., 2013).

In this study, we expanded this line of research by investigating the effect of *P. fluorescens* WCS417r on a second important generalist phloem-feeding model insect, the whitefly *Bemisia tabaci* (Hemiptera: Aleyrodidae). *Bemisia tabaci* is an important insect pest in many tropical and subtropical regions worldwide. It infests more than 600 plant species including important edible members of the Solanaceae family (Oliveira et al., 2001). *Bemisia tabaci* causes chlorosis on infested leaves, promotes the growth of sooty mold due to honeydew excretion (Schuster et al., 1996), and can transmit more than 200 species of plant viruses (Navas-Castillo et al., 2011). Like other phloem feeders, *B. tabaci* uses highly modified mouthparts (stylets) to intercellularly navigate through the plant cuticle, epidermis, and mesophyll and establish feeding sites in phloem sieve elements, causing comparatively little tissue damage (Tjallingii and Esch, 1993; Walker and Perring, 1994).

Our experimental system utilized tomato (*Solanum lycopersicum*) plants pre-inoculated with *P. fluorescens* WCS417r. We hypothesized that the relationship between the players in the *B. tabaci*—tomato—*P. fluorescens* system will add a general perspective to the complexity reported for the *M. persicae*—*A. thaliana*—*P. fluorescens* system (Pineda et al., 2012) due to two main reasons: (I) In *A. thaliana*, *B. tabaci* and likely other phloem feeders, are capable of manipulating plant-induced resistance (Zhu-Salzman et al., 2004; De Vos et al., 2007b; Kempema et al., 2007; Kusnierczyk et al., 2008). For example, *B. tabaci* infestation of *A. thaliana* plants was shown to induce the SA-responsive gene transcripts, while JA and ET—responsive gene transcripts were repressed or unchanged (Kempema et al., 2007). In contrast, *B. tabaci* feeding on plants like tomato and pepper (Solanaceae family), was shown to induce transcriptional changes in genes involved both in the JA/ET and SA pathways (Valenzuela-Soto et al., 2010; Yang et al., 2011); (II) Work with *A. thaliana* mutants indicated that *B. tabaci* is likely to be highly susceptible to JA-defenses, as mutants that activate JA defenses (*cev1*) or impair SA defenses (*npr1*, *NahG*) slowed *B. tabaci* nymphal development (Zarate et al., 2007).

Our hypothesis was tested through three main approaches: First, we determined if pre-inoculation of tomato plants with *P. fluorescens* WCS417r alters *B. tabaci* developmental and reproductive performance. Second, we examined the effect of pre-inoculation of tomato plants with *P. fluorescens* on the basal and induced expression levels of the JA/ET and SA defense pathways, before and during 12 days of *B. tabaci* infestation. Third, we determined the effect of pre-inoculation with *P. fluorescens* on tomato plant nutritional quality and the microbial composition of the rhizosphere.

## Materials and methods

### Plant material

Seeds of tomato plants (*Solanum lycopersicum* cv. H7998) were sown in sowing tray containing soil mixture (Tuff Merom Golan) that was autoclaved twice with a 24 h interval. After root inoculation, seedlings were transferred to 11 cm diameter pots containing the same soil mixture. Plants were maintained in Plexiglas cages with 50 mesh nets under growing conditions of 26 ± 2°C and long-day photoperiod conditions (L:D 14 h:10 h).

### Insect rearing

The *B. tabaci* Asia Minor—Middle East 1 (B) colony was collected from a melon field at the western Negev of Israel in 2003. Since its collection, the colony was maintained in the laboratory on Acala cotton (*Gossypium hirsutum* L cv. Acala), without exposure to insecticides, under standard greenhouse conditions of 14:10 L:D photoperiod, 26 ± 2°C. The homogeneity and purity of the colony was verified every 2–3 generations by cleaved amplified polymorphic sequences (CAPS) of the COI gene (Khasdan et al., 2005).

### Bacterial inoculation

*Pseudomonas fluorescens* WCS417r (Pieterse et al., 1996) was grown for 48 h at 28°C on King's medium B (King et al., 1954) supplemented with 25 μg of rifampicin per milliliter. Bacteria were collected and re-suspended in 0.1 M MgSO_4_ to achieve a final density of 10^8^ CFU ml^−1^ (OD_660_ = 0.1). Two-weeks-old tomato seedlings in sowing trays were dipped into the bacterial solution for 2 h. Control plants were dipped into 0.1 M MgSO_4_ solution. Two days post inoculation, seedlings were transferred into pots. After 1 week, inoculated plants were watered with 25 ml of bacterial solution while control plants were watered with 0.1 M MgSO_4_ solution.

### *Bemisia tabaci* reproductive performance assays

All *B. tabaci* reproductive performance experiments were conducted in a temperature-controlled room (L:D 14 h:10 h, 26 ± 2°C). Twelve adult couples (male and female), 72 h after emergence, were collected into a clip cage, 3 cm in diameter, which was attached to the third true leaf of 3–4-week-old pre-inoculated or non-inoculated (control) tomato plants. Plants were inoculated with *P. fluorescens* WCS417r 2 weeks earlier, while non-inoculated plants were treated with 0.1 M MgSO_4_ solution. After a 24 h oviposition period, all adults were collected and eggs were allowed to develop for 17 days. The number of eggs on each leaf was recorded 5 days after infestation. The total number of progeny, their developmental stages (second through early fourth nymphs, late fourth nymphs, empty exuvia indicating emergence), and viability status (live/dead), were recorded after 17 days. The developmental rate was estimated by calculating the proportion of 1st—early 4th nymphs, late fourth nymphs (red-eye yellow 4th nymph) and empty exuvia on each plant, 17 days after infestation. The survival rate was calculated as the proportion of live nymphs from the total number of eggs oviposited on each plant. Each treatment was replicated ~30 times in three independent experiments. Differences in development (for each stage separately) and survival rates of *B. tabaci* between pre-inoculated and non-inoculated tomato plants were analyzed using a generalized linear model with a logit link and a binomial distribution. Difference in oviposition was tested for significance using a generalized linear model with a Poisson distribution and the logarithmic link function. Statistical significance was assumed at *p* = 0.05. All statistical analyses conducted in this paper (see below) were performed with JMP statistical software version 7.0.1 (SAS Institute, USA).

### Expression of JA/ET- and SA-regulated defense genes

To determine if pre-inoculation of tomato plant roots with *P. fluorescens* WCS417r modifies the plant induced systemic defenses toward *B. tabaci* infestation, we quantified, by quantitative real-time PCR (qRT-PCR), the expression level of JA/ET pathway gene markers *Proteinase inhibitor I* and *II* (*PI-I* and *PI-II*, respectively) (Puthoff et al., 2010; Abd El Rahman et al., 2012) and the SA pathway gene markers *Pathogenesis-related protein 2* and *5* (*PR-2* and *PR-5*, respectively) (Van Loon et al., 2006). The list of gene names, qRT-PCR primers and product sizes is provided in Table A1. In order to be capable of separating the bacterial effect from that of insect infestation, four different treatments were applied: (I) control plants, not inoculated with *P. fluorescens* and without *B. tabaci* infestation (P-/B- plants); (II) *P. fluorescens* pre-inoculated plants without *B. tabaci* infestation (P+/B− plants); (III) non-inoculated plants with *B. tabaci* infestation (P−/B+ plants); (IV) *P. fluorescens* pre-inoculated plants with *B. tabaci* infestation (P+/B+ plants). Experiments were conducted in a temperature-controlled room (L:D 14 h:10 h, 26 ± 2°C). Pre-inoculation and infestation protocols were identical to the ones described above. Non-inoculated plants were treated with 0.1 M MgSO_4_ solution. An empty clip cage was attached to the third true leaf of 3–4-week-old un-infested plants.

Total RNA was extracted from 100 mg of third leaf tissue of plants that received one of the four aforementioned treatments. Samples were collected at four time points, representing different stages in the plant-insect-interaction cycle: prior to infestation (“day 0”—control), and one (“day 1”—the effect of 24 h of adults' feeding and egglaying), six (“day 6”—the beginning of egg hatching in which minimal effect of *B. tabaci* infestation is expected) and twelve (“day 12”—presence of *B. tabaci* 2nd and 3rd feeding nymphs—the effect of nymph infestation) days after *B. tabaci* infestation. Uninfested plants were treated in a similar way, and leaf samples were collected at the same time points as the *B. tabaci*-infested treatments. Three separate biological replicates were performed for each treatment in each of three independent experiments (total of 9 replicates per treatment). Each replicate consisted of one leaf from one plant that was sampled only once, in order to avoid the subsequent interfering effects of plant damage during leaf collection. Immediately after collection, leaf tissues were placed in liquid nitrogen and grounded by a tissuelyzer (Qaigen). RNA was extracted using Trizol reagent (Invitrogen), according to the manufacturer's instructions. RNA concentration and purity was measured by NanoDrop ND-100 (NanoDrop Technologies, USA) and 1 μl of 40 u/μl RNase inhibitor (Fermentas) was added to prevent RNA degradation. Two micrograms of total RNA were treated with DNase (Sigma) and RNA was stored at −80°C. cDNA synthesis was performed on 1 μg of total RNA using reverse transcriptase revertAID H minus (Ferments) and Oligo-dT primer (5′ TTTTTTTTTTTTTTTTTTTV 3′), according to manufacturer's instructions.

qRT-PCR reactions were performed using ABI 7300 (Applied Biosystems). Each reaction was optimally set to a master mix (18 μl) containing: 2 μl of cDNA, 150 nM of forward and reverse primers, 4.3 μl distilled deionized water (DDW) and 9 μl of Platinum SYBR Green qPCR SuperMix (invitrogen). PCR thermal conditions consisted of one cycle of 50°C for 2 min, one cycle of 95°C for 2 min, followed by 40 cycles of 95°C for 15 s and 60°C for 30 s. All the primers in this study were calibrated and adjusted to reaction conditions and showed slop values of 3.3–3.6 and *R*^2^ ≥ 0.98 in calibration curves. Reaction results were analyzed with the 7300 System SDS Software (Applied Biosystems). Quantification of the transcript levels were conducted according to the ΔCT method (Yuan et al., 2006) using *cyclopilin* (*CYP*) from tomato as the reference gene (Mascia et al., 2010). To minimize intra-experimental variation, each reaction was performed in triplicate of which ΔCT was calculated. Two negative controls were added to each reaction, a non-template control and a non-amplicon control (-RT control). Normalized ΔΔCT values were calculated by subtracting the mean ΔCT value of the P−/B− samples (see above) from the ΔCT values of the P+/B+, P+/B−,P−/B+ and P−/B− samples within each time point and experiment combination. This normalization was required both for excluding the effect of plant age on defense gene expression and for reducing the variance between experiments. Differences in ΔΔCT values were tested for significance by a Three-Way ANOVA model, in which bacteria (P+ or P−), insect (B+ or B−) and time period (0, 1, 6 or 12 days) were set as fixed effects. Specific means in the (bacteria X insect) interaction were selected *a priori* and orthogonal comparisons were conducted within each time period. Statistical significance was assumed at *p* ≤ 0.05.

### Carbohydrate extraction and quantification

Carbohydrate extraction was performed on tomato leaves, 2 weeks after *P. fluorescens* inoculation. Control plants were treated at the same time with 0.1 M MgSO_4_ solution. Third true leaf tissue material (100 mg) was placed in liquid nitrogen, grounded by a tissuelyzer (Qiagen) and extracted in 5 ml 80% (v/v) ethanol (30 min, 30°C). The extract was centrifuged (10 min, 2150 × g) and the pellet was extracted again with ethanol. After centrifugation, chlorophyll was removed from the soluble sugar supernatants by chloroform extraction. Samples were analyzed colorimetrically for soluble carbohydrates using the anthrone method (Yemm and Willis, 1954). Differences in soluble carbohydrate concentrations (mg/g leaf fresh weight) were tested for significance by a Two-Way ANOVA II model. Bacterial treatment was set as a fixed effect while experiment was set as a random effect. Statistical significance was assumed at *p* ≤ 0.05.

### Quantification of carbon (C) and nitrogen (N) contents

Quantifications of C and N contents were performed on two tissue sources: (I) tomato third true leaves, 2 weeks after *P. fluorescens* inoculation. Control non-inoculated plants were treated at the same time with 0.1 M MgSO_4_ solution. (II) Two hundred *B. tabaci* adults, 6 days after they infested *P. fluorescens* pre-inoculated plants or non-inoculated control plants. C and N contents were measured on samples of 2 mg dried and ground tissues (60°C, 48 h) using an elemental analyzer (Thermo-Electron Flash EA 1112) equipped with a copper/copper oxide column and a thermal conductivity detector. Differences in C and N contents (%) and C/N ratio were tested for significance (independently for plant and insect tissues) by a Two-Way ANOVA II model. Bacterial treatment was set as a fixed effect while experiment was set as a random effect. Statistical significance was assumed at *p* ≤ 0.05.

### Rhizobacterium DNA extraction and denaturing gradient gel electrophoresis (DGGE)

Rhizobacterium samples were collected from 0.2 g of soil that remained attached to the roots of the tomato plants after vigorous shaking. Samples were collected from four different treatments: (I) control plants, not inoculated with *P. fluorescens* and without *B. tabaci* infestation (P−/B− “day 0” samples); (II) *P. fluorescens* pre-inoculated plants without *B. tabaci* infestation (P+/B− “day 0” samples); (III) non-inoculated plants after 12 days of *B. tabaci* infestation (P−/B+ “day 12” samples); (IV) *P. fluorescens* pre-inoculated plants after 12 days of *B. tabaci* infestation (P+/B+ “day 12” samples). Three separate biological replicates were performed for each treatment. DNA extraction was conducted according to the manufacturer's instructions, using the Power Soil extraction kit (MoBio Laboratories, Inc., Carlsbad, CA) with one modification: to increase DNA concentration, the elution volume was set to 40 instead of 100 μl. The forward GC-clamp 968F and reverse 1401R primers, amplifying a ~450 bp product of bacterial 16S rRNA, were used in the PCR reactions (see primer's list in Table A1). PCR mixtures contained 10 μl Red load *Taq* master (Larova), 2 μl of each primer (10 pmol/μl), 3 μl template DNA and double-distilled water to a final volume of 50 μl. PCR thermal conditions consisted of denaturation at 94°C for 3 min, 35 cycles of 94°C for 30 s, 57°C for 30 s, and 72°C for 1 min, followed by 10 min at 72°C as an ending step. The presence of bacterial DNA products was verified by gel electrophoresis using 1% (w/v) agarose, stained with ethidium bromide (0.2 mg/ml). Denaturing gradient gel electrophoresis (DGGE) was performed according to Green et al. (2009). PCR products were loaded on a denaturing gradient gel containing 6% (w/v) polyacrylamide gel [acrylamide/bisacrylamide (37:1)] in 1 X Tris–acetate–EDTA (TAE) buffer with a 50–65% denaturing gradient (50–18% (v/v) formamide and 4.37 M urea, 65–26% (v/v) formamide and 5.86 M urea). Electrophoresis was performed with a Phor-U2 system (Ingeny, Goes, Holland) in 1 X TAE at 60°C, 95 V for 16 h. The gels were stained for 45 min. using 40 μl of Gel star (Lonza) in 300 ml 1 X TAE, followed by 15 min. rinsing in 1 X TAE. Visualization was performed using the AlphaImageri System (Alpha Innotech Corporation, California, USA). DGGE patterns were aligned using Fingerprinting® II software and the samples' densitometric curves were calculated. Analysis of similarity (ANOSIM) based on the Bray-Curtis distance matrix, calculated between the samples densitometric curves was performed. In addition, unweighted pair-group method with arithmetic mean (UPGMA) algorithm was applied to a Bray-Curtis distance matrix. Bootstrap values were calculated after running 10,000 replications. Analyses were conducted using the PAST software (http://folk.uio.no/ohammer/past/).

## Results

### Reproductive performance of *B. tabaci* on *P. fluorescens* WCS417r pre-inoculated and non-inoculated tomato plants

**Figure 1:**
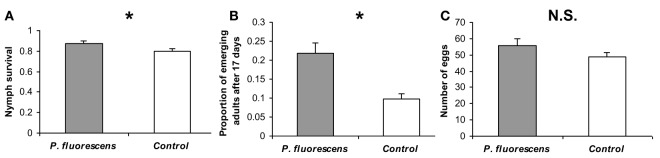
**Reproductive performance of *B. tabaci* on *P. fluorescens* WCS417r pre-inoculated and non-inoculated tomato plants**. **(A)** The proportion of live nymphs from the total number of eggs oviposited. **(B)** The proportion of progeny that had emerged as adults by day 17. **(C)** The mean number of eggs oviposited by 12 females during 24 h. Asterisks indicate significant differences (*P* ≤ 0.05). Errors bars represent standard error of the means (*n* = ~30). N.S. = Not significant.

*Bemisia tabaci* infestation was conducted 14 days after *P. fluorescens* WCS417r inoculation. At this time, the bacterial concentration in tomato roots was ~2.6 × 10^5^ CFU/g fresh-roots. Estimations of two independent life-history traits related to reproduction indicated increased nymphs performance on *P. fluorescens* pre-inoculated plants relative to non-inoculated control plants. Both the nymph survival rate and the proportion of progeny that emerged as adults, 17 days after oviposition, were significantly higher on pre-inoculated plants [χ^2^_(1)_ = 4.16, *p* = 0.041 and χ^2^_(1)_ = 11.09, *p* = 0.0009, respectively] (Figures 1A,B). Although the number of oviposited eggs was also higher on pre-inoculated plants than on non-inoculated plants, this difference was not significant [χ^2^_(1)_ = 1.68, *P* = 0.19] (Figure 1C).

Collectively, our performance experiments indicated that tomato plants, pre-inoculated with *P. fluorescens* WCS417r, are likely to be a more suitable host for *B. tabaci* than non-inoculated plants. In order to try gaining insight into this finding, we further explored three possibilities: (I) that pre-inoculated and non-inoculated plants differ in the activity levels of their induced resistance pathways; (II) that the two plant types differ in their nutritional status; (III) that *P. fluorescens* pre-inoculation manipulates the plant's quality (in terms of suitability for *B. tabaci*) through an indirect effect on the rhizosphere bacterial community.

### Characterizing the molecular activity of the JA/ET and SA pathways in tomato plants pre-inoculaed and non-inoculated with *P. fluorescens* WCS417r

qRT-PCR analyses were done to quantify the transcript levels of four marker genes: *PI-I* and *PI-II* (JA/ET-responsive genes) and *PR-2* and *PR-5* (SA-responsive genes) following *P. fluorescens* pre-inoculation or non-inoculation treatments. Analyses were conducted at four time points, representing different stages in the plant-insect-interaction cycle: “day 0” (before *B. tabaci* infestation), “day 1” (the effect of 24 h of adults' feeding and egglaying), “day 6” (the beginning of egg hatching in which minimal effect of *B. tabaci* infestation is expected) and “day 12” (presence of *B. tabaci* 2nd and 3rd feeding nymphs—the effect of nymph infestation) after *B. tabaci* infestation.

A striking phenomenon that was shared by all four genes analyzed, was the general lower activity levels of both the JA/ET and SA pathways in *P. fluorescens* pre-inoculated plants. This main effect was significant for the *PI-II*, *PR-2* and *PR-5* genes (*p* ≤ 0.040) and marginally significant also for *PI-I* (*p* = 0.063). The main effect of “time” was also highly significant for all four genes (*p* ≤ 0.036). All four genes (responding to the JA/ET or SA pathways) were significantly up-regulated at “day 1,” when compared to their expression levels at “day 6” (*p* ≤ 0.040), and the *PR-2* and *PR-5* genes were also significantly up-regulated at “day 1” when compared to “day 12” (*p* ≤ 0.0001). None of the four genes significantly differed in their expression levels between “day 6” and “day 12” although the *PI-I*, *PI-II* (up-regulated) and *PR-5* (down-regulated) genes were on the boarder of significance (*p* = 0.078, *p* = 0.094 and *p* = 0.057, respectively). In contrast, the main effect of “insect infestation” was found to be significant for only one gene marker (*PI-II*, *p* ≤ 0.008), where the expression level was higher in infested plants. Main effect statistical details are presented in Table A2.

Detailed analyses were conducted for each infestation period separately (summarized in Table 1; see Material and Methods for explanation on the four combinations of *P. fluorescens* WCS417r and *B. tabaci* treatments: P−/B−, P+/B−, P−/B+ and P+/B+). At “day 0,” no differences were observed, in the expression levels of all four analyzed genes, between the P−/B− and P+/B− tomato plants (*p* ≥ 0.20). At “day 1,” the expression levels of the *PR-2* and *PR-5* genes were significantly up-regulated in the (P−/B+) treatment when compared to the P−/B− treatment (*p* = 0.008 and *p* = 0.020, respectively), while the *PI-I* and *PI-II* genes showed similar non-significant trends (*p* = 0.095 and *p* = 0.14, respectively). In contrast, no differences were observed between the four genes' expression levels in the P+/B− and P+/B+ treatments (*p* ≥ 0.28), indicating that rhizobacteria colonization can likely reduce *B. tabaci*-dependent up-regulation of both SA- and JA/ET-responsive genes. The main finding at “day 6” was the reduced activity of both *PI-II* and *PR-2* (*p* = 0.037 and *p* = 0.017, respectively) in the P+/B+ treatment when compared to the P−/B+ treatment. In addition, a similar non-significant trend was observed in the expression pattern of *PI-I* (*p* = 0.20). *PI-II* also showed marginally–significant reduced expression-level in the P+/B− treatment when compared to the P−/B− treatment (*p* = 0.07), indicating a putative general reduced activity of the JA/ET pathway in *P. fluorescens* pre-inoculated plants at this time point. At “day 12,” non-significant differences were observed between the four treatment combinations of *P. fluorescens* and *B. tabaci* when the expression levels of the *PI-I*, *PR-2* and *PR-5* genes were compared (*p* ≥ 0.09). In contrast, the expression level of the *PI-II* gene was significantly up-regulated in the *B. tabaci* infested treatments (P−/B+ and P+/B+) when compared to the P+/B− treatment (*p* = 0.032 and *p* = 0.022, respectively), indicating the strong induced expression of the JA/ET pathway, during long period of *B. tabaci* infestation.

**Table 1:**
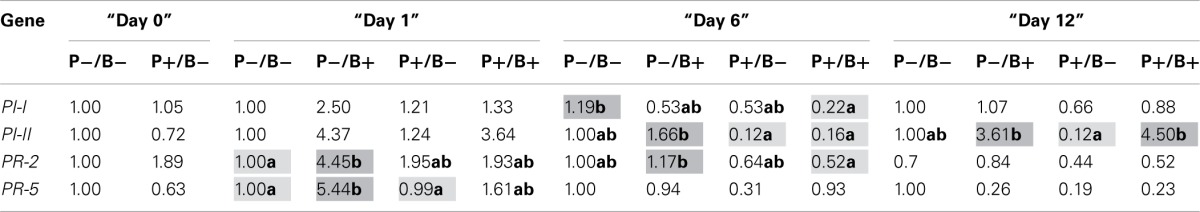
**Transcriptional profiles of four gene markers: *PI-I* and *PI-II* (JA/ET-responsive genes) and *PR-2* and *PR-5* (SA-responsive genes) in *P. fluorescens* pre-inoculation (P+) and non-inoculated (P−) tomato plants, with or without (control) *B. tabaci* infestation (B+ and B−, respectively)**.

*Samples were collected at four time points: “day 0” (prior to B. tabaci infestation and egglaying), “day 1” (the effect of 24 h of adults' infestation and egglaying), “day 6” (the beginning of egg hatching—minimal effect of B. tabaci infestation) and “day 12” (presence of B. tabaci 2nd and 3rd feeding nymphs—the effect of nymph infestation). Numbers indicate relative expression levels (2^−ΔΔ^CT). Normalized ΔΔCT values were calculated by subtracting the mean ΔCT value of the P−/B− samples (see above) from the ΔCT values of the P−/B+, P+/B−,P+/B+ and P−/B− samples within each time point and experiment combination. Different letters and gray shading (light vs. dark) within each time point and gene combination, indicate significant differences between the treatments (P ≤ 0.05)*.

### Plant and insect nutrient contents

In these experiments, we measured the levels, in *P. fluorescens* pre-inoculated and non-inoculated plants, of three chemicals thought to be important nutrients for arthropod herbivores: soluble carbohydrates, N (nitrogen), and C (carbon). N and C contents were also measured in *B. tabaci* adults, after 6 days of feeding on pre- or non-inoculated plants. Soluble carbohydrate concentrations and N and C contents were determined in dried and ground material of third true leaves. Pre-inoculated plant leaf tissues contained significantly more soluble carbohydrates [*F*_(1,47)_ = 4.15, *p* = 0.047] (Table 2) than non-inoculated plants. In contrast, both N and C contents were significantly higher in non-inoculated plants than in pre-inoculated plants [*F*_(1,10)_ = 5.92, *p* = 0.035 and *F*_(1,9)_ = 7.56, *P* = 0.022, respectively] (Table 2). Consequently, the C/N ratios were found to be similar in pre- and non-inoculated tomato plants [*F*_(1,10)_ = 0.29, *p* = 0.60] (Table 2). No significant differences were detected in the N and C contents and their C/N ratio, between *B. tabaci* adults that fed for 6 days on pre- and non-inoculated tomato plants (*p ≥ 0.13)* (Table 3).

**Table 2:** **The levels of soluble carbohydrates, N (nitrogen) and C (carbon) in *P. fluorescens* WCS417r pre-inoculated and non-inoculated plants**.

**Plant treatment**	**Nutritional content**			
	**Soluble carbohydrates (mg/g leaf FW ± SE)**	**N % (DW ± SE)**	**C % (DW ± SE)**	**C/N Ratio**
*P. fluorescens* WCS417r	2.49 ± 0.15	6.08 ± 0.05	41.11 ± 0.51	6.76 ± 0.06
Control	2.14 ± 0.12	6.27 ± 0.06	42.82 ± 0.54	6.83 ± 0.12
*P*-value	0.047	0.035	0.023	0.60

*FW, fresh weight; DW, dry weight; SE, standard error. Differences were tested for significance by a Two-Way ANOVA II model. Statistical significance was assumed at P ≤ 0.05*.

**Table 3:** **The N (nitrogen), and C (carbon) contents of *B. tabaci* adults, after 6 days of feeding on *P. fluorescens* WCS417r pre- or non-inoculated plants**.

**Plant treatment**	**Nutritional Content**		
	**N % (DW ± SE)**	**C % (DW ± SE)**	**C/N Ratio**
*P. fluorescens* WCS417r	9.60 ± 0.15	53.05 ± 0.37	5.53 ± 0.07
Control	9.34 ± 0.03	52.88 ± 0.53	5.66 ± 0.06
*P*-value	0.20	0.52	0.13

*DW, dry weight; SE, standard error. Differences were tested for significance by a Two-Way ANOVA II model. Statistical significance was assumed at P = 0.05*.

### DGGE profile of bacterial communities in the tomato rhizospere

**Figure 2:**
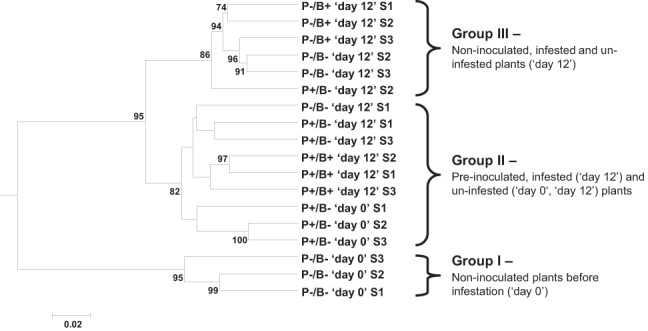
**Cluster analysis of tomato rhizosphere bacterial community patterns**. DGGE patterns were aligned using Fingerprinting® II software and the samples' densitometric curves were extracted. Unweighted pair-group method with arithmetic mean (UPGMA) algorithm was applied to a Bray-Curtis distance matrix calculated between the samples densitometric curves. Numbers at the nodes represent bootstrap values performed with 10,000 replications (bootstrap values >70 are presented). P−, non-inoculated control; P+, *P. fluorescens*-inoculated; B+, *B. tabaci*-infested plants; B−, uninfested control; “day 0,” prior to infestation; “day 12,” 12 days after infestation. S, sample.

The DGGE profiles of the bacterial communities of the tomato rhizosphere were analyzed by calculating the samples' densitometric curves. The *P. fluorescens* WCS417r bacteria sample, inserted as a control strain, could not be associated with high confidence, with any of the observed profiles, likely due to the low relative abundance of these bacteria (~2.6 × 10^5^ CFU/g fresh-roots) in the rhizosphere bacterial community. The ANOSIM test, performed on the densitometric curve matrix, indicated significant effects for the “time” (“day 0” or “day 12”) and “bacteria” (pre-inoculated or non-inoculated) treatments (*p* = 0.0004 and *p* = 0.015, respectively) but not for the “insect” (infested or un-infested) treatment (*p* = 0.38). Cluster analysis (UPGMA algorithm applied to the Bray–Curtis distance matrix) using 10,000 bootstrap repetitions, robustly divided the 18 samples (with two exceptions) into three main groups (Figure 2): group I contained the P−/B− “day 0” samples with similarity ≥ 93.3%; group II was formed by two clusters, one of P+/B− “day 0” samples with similarity of ≥ 94.1%, and the other of the P+/B− “day 12” and P+/B+ “day 12” samples with similarity ≥ 93.7%; group III contained P−/B+ and P−/B− “day 12” samples, with similarity ≥ 94.9%.

## Discussion

In the current study, we investigated the effects of pre-inoculation of tomato plants with the PGPR *P. fluorescens* WCS417r, on the performance of a generalist phloem-feeding model insect, the whitefly *Bemisia tabaci*. Based on the reported susceptibility of *B. tabaci* to JA-dependent defenses (Zarate et al., 2007) and the ability of *P. fluorescens* WCS417r to prime for enhanced JA-dependent resistance against generalists chewing insects (Van Oosten et al., 2008) and necrotrophic pathogens (Pieterse et al., 1998), we hypothesized that *P. fluorescens* WCS417r pre-inoculated tomato plants will resist *B. tabaci* infestation by strong expression of JA-responsive genes upon insect attack. On the contrary, our findings indicated a positive effect of pre-inoculation on *B. tabaci* performance, which was emphasized both by faster developmental rate and higher survivability of nymph stages on pre-inoculated plants. These unexpected findings are in-line with an independent study, published during our research, which reported that strain WCS417r has a positive effect on the performance (weight gain and intrinsic rate of increase) of the generalist aphid *M. persicae* when feeding on *A. thaliana* (Pineda et al., 2012). Collectively, these studies suggest a possible generalized positive relationship between *P. fluorescens* WCS417r and generalist phloem-feeders which is independent of the plant host.

Why do generalist phloem feeders respond differently than other insect herbivores to pre-inoculation of plant hosts with *P. fluorescens* WCS417r? Three possible explanations already exist in the literature. First, phloem feeders in general and *B. tabaci* in particular, are known to cause minimal damage to plant tissues while feeding (Johnson and Walker, 1999). This might enable them to avoid both constitutive and PGPR-primed induced plant-defense systems that require tissue damage (Kaloshian and Walling, 2005; Walling, 2008). Second, phloem feeders experience only plant defensive compounds that are translocated through the phloem, and are only marginally affected by those expressed in other tissues (Ramsey et al., 2010). Third, in *A. thaliana*, accumulating evidence suggest that phloem feeding insects such as aphids and whiteflies, can manipulate plant-induced resistance and are able to suppress effective JA defenses by the induction of the inefficient SA signaling-based responses (De Vos et al., 2007b; Kempema et al., 2007; Kusnierczyk et al., 2008).

However, these three classical explanations do not take into account a fourth unique characteristic of both the *M. persicae* and *B. tabaci* systems: the rhizobacteria-dependent suppression of genes related to the JA/ET defensive pathways (Pineda et al., 2012; this study). In the *M. persicae*—*A. thaliana*—*P. fluorescens* WCS417r reported system (Pineda et al., 2012), the presence of the rhizobacteria suppressed the expression of both *ABA1* (ABA-signaling responsive gene) and *MYC2* that were induced in plants infested with *M. persicae*, indicating a putative bacterial-dependent suppression of the MYC2-branch of the JA pathway. In the same system, the *PDF1.2* gene (responsive both to JA and ET) showed enhanced expression levels only at early infestation time periods (up to 24 h), both in non-inoculated and pre-inoculated aphid-infested plants. It was also recently reported that application of *P. rapae* (specialist chewing insect herbivore) larval oral secretion into wounded *A. thaliana* leaf tissue stimulated the ERF-branch of the JA pathway, but feeding of the larvae activated *MYC2* transcription which lowered the attractiveness of the leaf for insect feeding (Verhage et al., 2011). Collectively, these findings raise the possibility that *P. fluorescens* WCS417r pre-inoculation increases the performance of phloem-feeders by down-regulating efficient defenses related to the JA-pathway.

It is important to note, however, that the setting of our *B. tabaci*—tomato—*P. fluorescens* WCS417r system was somewhat different from that described for *M. persicae*, because the effects of adults (“day 1”) and nymphs (“day 12”) infestation were separated by collecting the adults after one day. More importantly, as *B. tabaci* eggs do not feed (the egg pedicel serves only as a conduit for water movement into the egg; Byrne et al., 1990), “day 6,” in which the eggs only begin to hatch, likely served as an intermediate buffering interval, where the system was dominated by the effect of *P. fluorescens* WCS417r pre-inoculation. At this time point, significant reduced activity of both *PI-II* and *PR-2* was observed in P+/B+ (pre-inoculated, infested) plants when compared to P−/B+ (non-inoculated, infested) plants and a similar non-significant trend was also observed in the expression pattern of *PI-I*. Interestingly, it was recently reported that *Helicoverpa zea* eggs triggered the expression of the *PI-II* gene at the ovipsotion site in tomato (Kim et al., 2012), which might partially explain the higher (although not significant) expression of *PI-II* in the P−/B+ (non-inoculated, infested) treatment relative to the P−/B− (non-inoculated, un-infested) treatment at “day 6.” In addition, the *PI-II* gene also showed a marginally-significant reduced expression level in P+/B− (pre-inoculated, un-infested) plants when compared to P−/B− (non-inoculated, un-infested) plants. In tomato, the *PI-I* gene is induced by wounding, systemin and JA treatments while *PI-II* requires both ET and JA signaling for its induction (Rojo et al., 2003). Moreover, the *PR-2* gene can be positively regulated not only by SA but also by ET (Van Kan et al., 1995). Taken together, these findings suggest a possible down-regulation of both ET-responsive genes and JA-responsive genes (to a lesser extent) in *P. fluorescens* WCS417r pre-inoculated tomato plants, which is in line with a previous report of significant reduction in the expression of genes encoding ET-related transcription factors in roots of *A. thaliana* plants pre-inoculated with *P. fluorescens* WCS417r (Verhagen et al., 2004). Moreover, it was recently hypothesized that PGPR modulate host immune responses by interfering with the ET signaling pathway (Zamioudis and Pieterse, 2012). The suppressed expression pattern of the JA/ET-pathway at “day 6,” the exact time when eggs hatch and young nymphs are most susceptible to plant defenses, might partially explain the better nymph performance observed on pre-inoculated plants relative to non-inoculated plants. However, it is also clear that a better characterization of the mechanisms by which *P. fluorescens* WCS417r interacts with tomato and other plants, is a prerequisite for a better understanding of the subsequent effects on the interactions of the plants with phloem-feeding insects (Pineda et al., 2013).

In contrast to the above, the expression patterns of the *PI-I*, *PI-II*, *PR-2*, and *PR-5* genes at “day 1” and “day 12” were largely affected by adult feeding/egglaying and nymph feeding, respectively, which seemed to override the PGPR repressing effect. Similar to previous findings in other Solanaceae systems (Sanchez-Hernandez et al., 2006; Estrada-Hernandez et al., 2009; Puthoff et al., 2010; Valenzuela-Soto et al., 2010; Yang et al., 2011), the response of plants to *B. tabaci* feeding, involves up-regulation of both SA- and JA/ET-responsive genes. This is in contrast to the reported changes in gene expression during *B. tabaci* infestation of *A. thaliana*, where SA-responsive genes were found to accumulate while JA/ET-responsive wound/defense genes were found to be suppressed or unchanged (Kempema et al., 2007; Zarate et al., 2007). Previously, Sanchez-Hernandez et al. (2006) has shown that nymph development of *B. tabaci* is delayed on a transgenic line that ectopically express prosystemin (*35S:ProSys*), while Valenzuela-Soto et al. (2010) observed enhanced nymph development on tomato *spr2* (*suppressor of prosystemin-mediated responses2*) mutants that were compromised in linolenic acid (LA) and JA synthesis. Collectively, two conclusions can be made: (I) avoiding activation of the JA/ET-response pathway in tomato would be advantageous to *B. tabaci* success; (II) in contrast to *A. thaliana*, tomato plants perceive *B. tabaci* infestation more like a nectrotrophic pathogen than tissue-damaging insect herbivores or biotrophic pathogens and activate the putatively “right” defensive pathways to tolerate the insect.

Besides promoting plant growth indirectly by protecting plants from diseases caused by different types of pathogens or herbivore insects, beneficial soil-borne bacteria can also promote plant growth directly. For example, *Rhizobium* bacteria (as well as other bacteria from related genera), establish intimate symbiotic relationship with leguminous plants that involves fixation of atmospheric nitrogen for the benefit of the plant (Spaink, 2000). Mycorrhizal fungi form symbiotic associations with the roots of the majority of herbaceous plant species in terrestrial systems. The fungus is thought to gain supply of carbon, transferred from the host plant, while the plant benefits from an enhanced nutrient uptake (Fitter, 1990). In addition, improved root development and water and mineral uptake have been widely reported in various plant species following inoculation with the PGPR *Azospirillum brasilense* (Fibach-Paldi et al., 2012). Although root colonization by *P. fluorescens* WCS417r was mostly associated with an increase in plant defensive capacity (Van Loon, 2007), our analyses indicated that the nutrient contents of tomato leaf tissues were affected by the presence of *P. fluorescens* in the rhizospere. Pre-inoculated plant leaf tissues contained significantly more soluble carbohydrates, while both N and C contents were significantly higher in non-inoculated plants. The C/N ratios, on the other hand, were found to be similar in pre- and non-inoculated tomato plants. As a phloem-feeder, *B. tabaci* ingests a diet that is rich in soluble carbohydrates (Hendrix et al., 1992). Although it is unlikely that soluble carbohydrates concentrations were a limiting factor in our system, their 15% increase in pre-inoculated plants, might have changed the insect's diet from “rich” to “richer,” partially explaining the better nymph performance on these plants. On the other hand, plant phloem sap is generally considered to be limited in N (Byrne and Miller, 1990). However, *B. tabaci* and other phloem-feeding insects, such as aphids, were shown to excrete amino acids (Isaacs et al., 1998). Moreover, *B. tabaci* can rapidly sense and metabolically adjust to altered plant N status (Crafts-Brandner, 2002). As the tomato plants in our system were well fertilized, we find it improbable that the differences found in N content between pre-inoculated and non-inoculated plants, affected the insect's developmental performance. Moreover, no significant differences were detected in the N and C contents and their C/N ratio, between *B. tabaci* adults that fed for 6 days on pre- or non-inoculated tomato plants. Similarly, *M. persicae* exhibited improved growth and reproduction on *Plantago lanceolata* plants infected with arbuscular mycorrhizal fungi, despite the fact that the plants had lower N contents (Gange and West, 1994). The higher C and N contents in non-inoculated plants could also lead to an increase in the production of nitrogen- and carbon-based plant defenses. However, this possibility was not further explored in our study.

Last, we tested whether the enhanced performance of *B. tabaci* on *P. fluorescens* WCS417r pre-inoculated tomato plants could be associated with the ability of the rhizobacteria to affect the soil bacterial community. Both the ANOSIM and cluster analyses, performed on the densitometric curves of the DGGE profiles, indicated a significant effect on the rhizosphere bacterial community, for the presence of the bacteria, and a high similarity between pre-inoculated plant samples, regardless of their infestation state or the length of the infestation period. It is important to clarify that although in these experiments, we used sterilized soil, the experiments were not performed under sterile conditions. Therefore, it is likely that under the experimental conditions, rhizosphere bacteria arrived with the seeds (and with seedlings upon transfer to pots), or from both irrigation water and air. Since we used a sterilized soil, bacteria coming from these sources could highly proliferate during the experiment, due to the biological vacuum caused by soil sterilization. Beneficial rhizobacteria can manipulate the plant's microbiome by suppressing the activity of pathogenic and non-pathogenic microorganisms not only by activating ISR but also through microbial antagonism (Van Loon, 2007). This is largely achieved by mechanisms such as competition for both nutrients and space and antibiosis through the production of specific and non-specific microbial metabolites, lytic enzymes, volatile compounds or other microbial toxins (Haas and Defago, 2005; Doornbos et al., 2012). However, the net effect of these below-ground interactions on the plant's nutritional quality and defense status above-ground, and their direct impact on phloem-feeders longevity, fecundity and nymph development is yet to be determined.

In conclusion, the development of environmentally friendly pest control tactics that are based on soil beneficial organisms can extend the range of options for maintaining the health and yield of crops. PGPR inoculants (*Azospirillum*, *Bacillu*s, *Pseudomona*s, etc.) are now available for a variety of crops (Hayat et al., 2010; Helman et al., 2011). Moreover, previous studies on *P. fluorescens* have established that soil inoculation can give protection against several important lepidopteran agricultural pests (Pineda et al., 2010), making the technology a future “green” alternative strategy to chemical insecticides. Our findings, however, suggest that the technology might not be applicable for controlling phloem-feeding pests, which are expected to benefit from its implementation into agricultural systems.

### Conflict of interest statement

The authors declare that the research was conducted in the absence of any commercial or financial relationships that could be construed as a potential conflict of interest.

## Appendix

**Table A1:** **Quantitative real-time PCR (qRT-PCR) and denaturing gradient gel electrophoresis (DDGE) list of forward (F) and reverse (R) primers and PCR product sizes**.

**Gene**	**Primer direction**	**Sequence**	**Product size (bp)**
*CYP*	F	5′-GAGTGGCTCAACGGAAAGCA-3′	80
	R	5′-CCAACAGCCTCTGCCTTCTTA-3′	
*PI- II*	F	5′-TTCAATTTGGCGTCGGTGGC-3′	95
	R	5′-TCACTCTCTCCTTCACATAC-3′	
*PI -I*	F	5′-CCAGTGGAAGATGATGGAGA-3′	221
	R	5′-GGGAGTAACTCGAACAATGC-3′	
*PR2*	F	5′-CGAGATGGTGGGTACAGAAGAAC- 3′	108
	R	5′-CAAGATTGGAAGTGCCAGTAACAG G-3′	
*PR5*	F	5′-AAACGGTGAATGCCCTGGTTCA-3′	102
	R	5′-AGGACCACATGGACCGTGATTA-3′	
*16S GC- clamp*	F	GC clamp-5′-AACGCGAAGAACCTTAC-3′	~450
	R	5′-CGGTGTGTACAAGGCCCGGGAACG-3′

**Table A2:** **ANOVA results (*F*-values and significance) of quantitative real-time PCR (qRT-PCR) analyses (main effects)**.

**Source**	***PI-I***		***PI-II***		***PR-2***		***PR-5***	
	***F*-value**	***P*-value**	***F*-value**	***P*-value**	***F*-value**	***P*-value**	***F*-value**	***P*-value**
“Bacteria”	3.55_ (1, 82)_	0.063	4.40_ (1, 80)_	0.039^*^	4.57_ (1, 73)_	0.036^*^	4.37_ (1, 81)_	0.040^*^
“Time”	5.79_ (2, 82)_	0.004^*^	3.45_ (2, 80)_	0.036^*^	16.53_ (2, 73)_	<0.0001^*^	8.53_ (2, 81)_	0.0004^*^
“Insect Infestation”	0.050_ (1, 82)_	0.824	7.47^*^_ (1, 80)_	0.008^*^	2.63_ (1, 73)_	0.110	1.04_ (1, 81)_	0.311

*See Material and Methods for detailed description of applied model. Asterisks indicate statistical significance (P ≤ 0.05)*.
